# Low molecular weight heparin use in unexplained recurrent miscarriage

**DOI:** 10.12669/pjms.306.5477

**Published:** 2014

**Authors:** Halide Yuksel, Semra Kayatas, Aysen Telce Boza, Murat Api, A. Aktug Ertekin, Cetin Cam

**Affiliations:** 1 Halide Yuksel, Zeynep Kamil Women and Children Diseases Training and Research Hospital, Department of Obstetrics and Gynecology, Istanbul, Turkey, 34668.; 2 Semra Kayatas, Zeynep Kamil Women and Children Diseases Training and Research Hospital, Department of Obstetrics and Gynecology, Istanbul, Turkey, 34668.; 3 Aysen Telce Boza, Zeynep Kamil Women and Children Diseases Training and Research Hospital, Department of Obstetrics and Gynecology, Istanbul, Turkey, 34668.; 4 Murat Api, Zeynep Kamil Women and Children Diseases Training and Research Hospital, Department of Obstetrics and Gynecology, Istanbul, Turkey, 34668.; 5 A.Aktug Ertekin, Zeynep Kamil Women and Children Diseases Training and Research Hospital, Department of Obstetrics and Gynecology, Istanbul, Turkey, 34668.; 6 Cetin Cam, Zeynep Kamil Women and Children Diseases Training and Research Hospital, Department of Obstetrics and Gynecology, Istanbul, Turkey, 34668.

**Keywords:** Enoxaparin, Tinzaparin, Live birth rate, Unexplained recurrent miscarriage

## Abstract

***Objective:*** The aim of the study was to investigate whether the use of low molecular weight heparin (LMWH) improve live birth rates when compared with control group in patients with unexplained recurrent miscarriages (URM).

***Methods:*** In this prospective observational study 150 women with a history of two or more previous unexplained first trimester pregnancy loss who received LMWH; either enoxaparin (n=50), tinzaparin (n=50) or nothing (n=50) were followed for the pregnancy outcome measures. Only the patients who have used standardized dosage of LMWH (4000 IU/day enoxaparin or 3500 IU/day tinzaparin ) were included to the study. The primary end point was the live birth rate and secondary end points were the side effects, late pregnancy complications and neonatal outcome in the study cohorts.

***Results:*** Live birth was achieved 85% of the LMWH group and 66% of the control group (p=0.007). According to the subgroup analysis; live birth rates did not differ significantly between the enoxaparin and tinzaparin group (84% and 86%, respectively). Maternal and neonatal side effects were not statistically significant among the study participants.

***Conclusion:*** Thromboprophylaxis with LMWH resulted in a improved live-birth rate in patient with 2 or more consecutive unexplained recurrent pregnancy loss. Nevertheless these findings need to be confirmed in larger randomized trials.

## INTRODUCTION

Recurrent miscarriage is defined as two or more consecutive pregnancy losses before 20 weeks of gestation is a common health problem affecting 1–5% of women at reproductive age and the etiology of 68% of recurrent abortions is unknown. In the absence of any therapeutic intervention, only about 25% of these pregnancies results in live births.^1^ According to some authors thrombophilic markers are not the only criteria for the initiation of thromboprophylactic treatment.^2^^,^^3^ Other investigators, however suggested not to treat unexplained miscarriage without evidenced antiphospholipid syndrome or inherited thrombophilia, with heparin or aspirin because of lack of evidence of any benefit and potential risks of therapy. The fact that thrombosis at placental level is a common finding whether antiphosholipid antibody are present or not, suggest that other pathologic mechanisms are also involved leading to same outcome, that is the fetal loss.^4^ Although in the literature there is no consensus regarding the benefit of antithrombotic therapy even in consecutive unexplained pregnancy losses,^5^ low molecular weight heparine (LMWH) is widely used as prophylaxis in recurrent miscarriages in general obstetric practice. The uncertain etiology and pathogenesis of URM have meant that treatment has remained empirical.

Enoxaparin is the most commonly used agent in the existing trials.^6^^,^^7^ Tinzaparin sodium is also a LMWH and, its biochemical and pharmacokinetic differences from enoxaparin may have clinically important effects.^8^ The data about the administration of tinzaparin during pregnancy is limited. However, LMWH alone throughout the pregnancy in patients with URM has not been sufficiently investigated and also, there is a lack of evidence for comparing LMWH molecules in these patients. In this observational study, we aimed to investigate whether the use of LMWH (either enoxaparin or tinzaparin) improves live birth rates when compared with control (without any thromboprophylaxis) group in women with URM. Secondly, we followed the safety measures of tinzaparin compared to enoxaparin in terms of prevention of late pregnancy complications, maternal and neonatal side effects despite LMWH do not cross the placenta.^9^

## METHODS

Women with URM in first trimester of their pregnancy who had already been under the treatment of LMWH (either enoxaparin or tinzaparin) or no treatment, aged between ≥ 18 and < 35 years, in our tertiary teaching hospital between March 2010 and January 2012 were followed till the end of their pregnancy. 

All women had normal results for parental karyotyping, hysterosalpingography or hysteroscopy, thyroid function and glucose tolerance tests, serum prolactin, homocysteine levels and mid-luteal progesteron level. All included patients have been screened for thrombophilia, all investigations were checked and patients with luteal phase defects, infections represented namely by Mycoplasma and Chlamydia, pathological levels for antinuclear factor or antiphospholipid antibodies (anticardiolipin IgG, IgM antibodies or lupus anticoagulant) and with hereditary thrombophilia patterns protein C, S and antithrombin deficiencies and presence of factor V Leiden mutation, prothrombin (G20210A) mutation and homozygosity for MTHFR (C677T) were not included to study. 

Women with cardiovascular disease, bleeding diathesis, previous thromboembolic phenomena, diabetes mellitus, vaginal bleeding, multiple pregnancy, smoking, morbid obesity and presence of contraindication for anticoagulant therapy were also excluded.

For each group (tinzaparin, enoxaparin and control group) fifty patients with unexplained recurrent pregnancy loss were included to the study. Totally 150 patients were followed throughout their pregnancy. Only the patients, who have used standardized dosage of the study medication of either 4000 IU/day in enoxaparin group or 3500 IU/day in tinzaparin group when fetal viability was confirmed by ultrasonography at 6 weeks of gestation were followed till the end of their pregnancy. Women receiving LMWH had been tought to self-inject subcutaneously in the anterolateral abdominal wall on the right and left sides alternatively. Adherence was confirmed with telephone interview biweekly for the first 4 weeks. Prenatal follow-up was obtained to assess fetal growth, fetal well-being and drug side effects for every 4 weeks until 32 weeks, every 2 weeks between 32-36 weeks and then weekly until delivery if pregnancy reaches to term. All pregnant women underwent genetic screening in the first trimester of pregnancy. Treatment was continued until abortion or delivery. Before planned induction of labor or elective cesarean delivery, LMWH treatment were ceased to prevent intraoperative hemorrhage. 

Study outcomes were listed as live birth rate, abortion rate, numbers of women with pre-eclampsia, IUGR, placental abruption and drug side effects as thrombocytopenia, thrombotic episodes, antepartum, postpartum bleeding, injection site hematoma, subcutaneous bruises and allergic skin reactions. Maternal platelets were checked at every visit for heparin induced thrombocytopenia (defined as a platelet count of <150,000 per cubic millimeter), bleeding episodes (i.e., bleeding from the gums or nose and the amount of vaginal blood loss at delivery) were recorded.

All infants were examined by a pediatrician after delivery. Perinatal outcomes in terms of birth weight, gestational week, number of neonates hospitalized in neonatal intensive care unit (NICU), neonatal mortality, neonatal bleeding and congenital anomalies were evaluated. 

The study protocol was conducted according to the revised Declaration of Helsinki and was approved by the local Research and Ethics Committee (Ethics Committee number 52). Written informed consents from all subjects who accepted to take place in this trial were obtained. 

The primary efficacy endpoint was live birth rate and secondary efficacy endpoints were late pregnancy complications as preeclampsia, intrauterine growth restriction (IUGR) and placental abruption. Safety endpoints were maternal and neonatal drug related adverse events.

***Statistics: *** All statistical analyses were performed with the Statistical Package for the Social Sciences version 15.0 (SPSS Inc, Chicago, IL, USA). Differences between the means in normally distrubuted variables were compared by using Student’s t-test. Chi-square test was performed on categoric variables. A p value of <0.05 was accepted as statistically significant.

## RESULTS

There were no differences among the groups in terms of age, body mass indices and the numbers of previous abortions (Table-I). A hundred women treated with LMWH had significantly lower abortion rates (15%) when compared with 17 control women (34%) (p=0.007). The rates of live births were 85% and 66% in LMWH and control groups (p=0.007). Also birth weeks were significantly lower in LMWH group when compared with control group 36.5±3.7 and 38.2 ± 1.9, respectively (p=0.001) (Table-II). Neonates born to the women with LMWH have similar birth weight with the control group (3185± 614g and 3251± 320g, respectively p=0.41).

Pregnancy complications are shown in Table-II. The incidence of preeclampsia in LMWH and control groups were 13% and 4%, respectively (p=0.08). IUGR was seen in one (2%) pregnancy in LMWH group and none of the pregnancies complicated with IUGR in control group. Placental abruption was not observed in any of the groups. Statistical analysis revealed that in terms of late obstetric complications there were no statistically significant differences among LMWH groups and control group.

Regarding the neonatal outcome; 4 neonates in LMWH group and one in control group needed to NICU (p=0.52). No instances of neonatal bleeding and congenital anomalies were reported. There was 5 neonatal death in LMWH group which occurred in babies born at 22, 24, 29 and 32 weeks of gestation because of preeclampsia and one in 28 weeks of gestation because of premature rupture of membranes. No neonatal death was observed in control group (p=0.10) (Table-II). 

According to the subgroup analysis; there were no statistically significant differences between both enoxaparine and tinzaparin groups as regards to rate of live birth (84% vs 86%) and the abortion rates (16% and 14%) (p=0.77). Birth weeks were significantly lower in enoxaparin group when compared to tinzaparin group (35.6±6.8 vs. 37.3±3.2, respectively, p=0.035) (Table-III).

Maternal side effects as antepartum or postpartum bleeding, heparin-induced thrombocytopenia, venous or arterial thrombotic episodes, hematoma due to regional anaesthesia or daily injection were not observed in any of the groups, in one case from enoxaparine group, subcutaneous bruises and in one case from tinzaparine group allergic local skin reaction was observed (Table-III). 

## DISCUSSION

It has been demonstrated that women with either congenital or acquired thrombophilia might benefit from LMWH in respect to live birth rates, abortion and late obstetrical complication rates.^10^^,^^11^ However, the use of LMWH to prevent recurrent miscarriages in thrombophilia remains controversial because of the small total number of women treated with LMWH in these trials (approximately 60), significant methodological problems as absence of control arms in study design. Large, well-designed randomized trials are needed. On the other hand, the management of women with a history of pregnancy loss without an identified cause is unclear and the role of anticoagulants for women with URM remains uncertain. Various treatment strategies have been tested. Most have focused on the use of thromboprophylaxis especially with enoxaparine alone or others have reported that combination treatment of prednisone, aspirin, folate and progesterone might be as effective treatment as enoxaparin alone.^12^ In a systematic review performed in women with a history of URM to determine a pooled risk ratio analysis for the effect of LMWH in achieving live birth could not performed because of the significant heterogenity observed among the studies.^13^

**Table-I:** Demographic characteristics of women included in our study**.**

	**LMWH **(n=100)	**Control **(n=50)	***p*** ** value**
Age , (years), mean ( ±sd)	28 (±5)	28.8 (±6)	0.40
BMI, kg/m², mean (±sd)	25.5 (±3)	25.7(±2.1)	0.64
Previous abortion count, median (range)	3 (2-4)	2 (2-5)	0.08

LMWH; low molecular weight heparin, BMI; body mass index, sd; standard deviation.

* **p<0.05**

**Table-II:** Comparison of groups in terms of fetal and neonatal outcome, maternal and neonatal safety.

	**LMWH **(n=100)	**Control **(n=50)	***p*** ** value**
Live birth n(%)	85 (85%)	33 (66%)	0.007*
Abortion rate n(%)	15 (15%)	17(34%)	0.007*
Abortion week, mean (±sd)	9.3 (±2.4)	9.24 (±1.4)	0.85
Birth week, mean (±sd)	36.5 (±3.7)	38.2 (±1.9)	<0.001*
Preeclampsia, n(%)	13 (13%)	2 (4%)	0.08
IUGR, n(%)	1 (2%)	0 (0%)	0.47
C/S rate, n(%)	42 (42%)	10 (20%)	0.06
Maternal safety outcome			
Subcutaneous bruises, n(%) Allergic skin reactions, n(%)	1(1%) 1(1%)	0(0%) 0(0%)	<0.001*
Neonatal outcome			
Birth weight (gram), mean(±sd)	3185 (±614)	3251 (±320)	0.41
Neonatal safety outcomes, n(%)			
Postpartum ex NICU	5 (5%) 4 (4%)	0 (0%) 1 (2%)	0.10 0.52
Premature birth, n(%)			
<32 birth week ≥32 birth week	24 (24%) 76 (76%)	18 (36%) 32 (64%)	0.12

LMWH; low molecular weight heparin, IUGR; intrauterine growth retardation,

C/S; cessarian section, NICU; neonatal intensive care unit, sd; standard deviation.

* **p<0.05**

**Table-III:** Subgroup analysis of tinzaparin and enoxaparin group in terms of fetal and neonatal outcome, maternal and neonatal safety.

	**Tinzaparin **(n=50)	**Enoxaparin **(n=50)	***p *** **value**
Live birth, n(%)	43 (86%)	42 (84%)	0.77
Abortion rate, n(%)	7 (14%)	8 (16%)	0.77
Abortion week, mean (±sd)	9 (±1)	9.69 (±3.37)	0.61
Birth week, mean ( ±sd)	37.3 (±3.2)	35.6 (±6.8)	0.035*
Preeclampsia, n(%)	5 (10%)	8 (16%)	0.37
IUGR, n(%)	0 (0%)	1 (2%)	0.31
C/S rate, n(%)	16 (32%)	26 (52%)	0.072
Maternal safety outcome, n(%)			
Subcutaneous bruises Allergic skin reactions	0 (0%) 1 (2%)	1 (2%) 0 (0%)	0.31 0.31
Neonatal outcome			
Birth weight,(gram), mean (±sd)	3212 (±672)	3149 (±554)	0.63
Neonatal safety outcomes, n(%)			
Postpartum ex NICU	1 (2%) 1 (2%)	4 (8%) 3 (6%)	0.16 0.30
Premature birth, n(%)			
<32 birth week ≥32 birth week	9 (18%) 41 (82%)	15 (30%) 35 (70%)	0.16 0.16

IUGR; intrauterine growth retardation, C/S; cessarian section,

NICU; neonatal intensive care unit, sd; standard deviation.

* **p<0.05**

A prospective randomized study about administration of enoxaparin and aspirin to unexplained recurrent miscarriage cases conducted by Dolitsky et al. One hundred and four women were randomized as 40 mg enoxaparine group and 100 mg aspirine group and as soon as fetal cardiac activity was seen prophylaxis was started and live birth rate was 81.5% in enoxaparine group and 84% in aspirine group.^7^ Fawzy et al. achieved a live birth rate of 81% using enoxaparin 20 mg a day in women with ≥3 fetal losses suffering from URM when compared with control group with a live birth rate of 48%.^12^

A modest improvement in outcomes as reducing early and late spontaneous abortions for women with recurrent pregnancy loss with unexplained etiology treated with LMWH over no treatment [live birth risk ratio 1.07, 95% CI 1.00–1.14] when given in first trimester and continued throughout pregnancy.^14^

On contrary, some investigators have reported no effects of enoxaparin and aspirine in live birth rates of women with unexplained recurrent miscarriages.^2^ In accordance with previously reported data, a multicenter randomized controlled trial of LMWH and low-dose aspirin plus intensive pregnancy surveillance resulted in a 22% miscarriage rate in women with URM versus 20% in the group receiving intensive surveillance alone.^5^ This study would thus suggest no basis for using LMWH or aspirin for URM. Recently, in a randomized, placebo controlled trial involving 364 women with URM assessed whether aspirin combined with LMWH nadroparin at a dose of 2850 IU or aspirin alone as compared with placebo would improve the live birth rate. This study concluded that neither aspirin combined with nadroparin nor aspirin alone improved the live birth rate as compared with placebo.^15^

The use of emprical therapy in women with URM is undoubtedly unneccessary in view of the fact that supportive care alone offers a chance of up to 75% for a successful pregnancy.^16^ However, there is a substantial amount of patients given LMWHs without adequate evidence and the prognosis of these patients were unkown. In the current study, LMWHs are found to be effective in improving live birth rate even in the absence of demonstrated ethiologic factors. This effect might be due to an array of properties of heparin other than its anticoagulant activity. Heparin has an anti-inflammatory effect that deciduas from women with recurrent miscarriages show common pathology that necrosis, acute and chronic inflammation and vascular thrombosis compared with those of women with normal pregnancies.^17^ Also heparin has an anti-complement effect which is absolutely required to prevent pregnancy loss and thrombosis.^18^^,^^19^

Recurrent pregnancy loss has been associated with a higher incidence of late obstetric complications.^20^ In Dolitzky et al. study these obstetric complications were not seen and they commented that either the obstetric complications are associated with thrombophilias or the treatment had a beneficial effect on both enoxaparin and aspirin group.^7^ In our study, preeclampsia and IUGR rate were higher in LMWH group when compared with control group but it was not statistically significant.

The subgroup analysis revealed that there were no statistically significant differences between both enoxaparin and tinzaparin group with respect to live birth rate and abortion rate. We think that both LMWHs have the group effect property. Tinzaparin has been shown to be safe and effective anticoagulant in the management of recurrent pregnancy loss in thrombophilic disorders.^7^ Both enoxaparin and tinzaparin are synthesized by depolymerization of unfractionated heparine. Enoxaparin is produced chemically whereas tinzaparin is an enzymatic product. As a result of this variation, these LMWH products may display differences in their relative inhibitory activities against factor Xa (FXa) and factor IIa (FIIa).^18^ It is not clear which effect is more important in inhibiting thrombosis.^21^^,^^22^ The clinical relevance of these biochemical and pharmacologic differences between LMWH molecules is uncertain. A study comparing the antithrombotic properties of enoxaparin, tinzaparin and deltaparin revealed significant differences in anti-FXa and anti-FIIa activity between products. The authors concluded that although some laboratory differences are present, no clinical differences between these products are found.^23^ It may be appropriate to carry out another study of enoxaparin and tinzaparin in unexplained pregnancy loss in a larger cohort of patients.

As a result of our study, the use of LMWH in the first trimester of pregnancy appears to be safe for mother and neonate; maternal bleeding, venous/arterial thrombotic episodes or heparin induced thrombocytopenia were not observed. Although our study is not large enough to make a final conclusion, with regard to neonatal complications, congenital anomalies and neonatal bleeding were not seen.

There were some limitations in the current study. The sample size was not determined prior at the beginning of the study to reach a proper power. The number of patients in each group was arbitrarily chosen however comparable to the previous studies. The treatment groups were also not a randomly assigned. So the potential biases were not excluded including; selection bias, tender loving care and close follow-up of patients who have been under the active treatment arms. 

In conclusion, with a limited number of participants, the present data demonstrates that in women with URM, thromboprophylaxis with LMWH is superior to no treatment. The need for larger well designed trials still remains.

## Author Contribution:

**SK, HY: **Conceived the study and did data collection. **AE, CC:** Designed the study and did review.

**SK:** Wrote the manuscript. **AB, MA:** Did review, statistical analysis and editing of manuscript.

## Notes

***Declaration of interest: ***The authors report no conflicts of interest. The authors alone are responsible for the content and writing of the paper.
